# The Effect of Alternating High-Sucrose and Sucrose Free-Diets, and Intermittent One-Day Fasting on the Estrous Cycle and Sex Hormones in Female Rats

**DOI:** 10.3390/nu14204350

**Published:** 2022-10-17

**Authors:** Joanna Sadowska, Wioleta Dudzińska, Izabela Dziaduch

**Affiliations:** 1 Department of Applied Microbiology and Human Nutrition Physiology, Faculty of Food Sciences and Fisheries, West Pomeranian University of Technology, ul. Papieża Pawła VI 3, 71-459 Szczecin, Poland; 2 Department of Physiology and Biochemistry, Institute of Biology, University of Szczecin, Felczaka 3c, 71-412 Szczecin, Poland; 3 Department of Functional Diagnostics and Physical Medicine, Pomeranian Medical University in Szczecin, Żołnierska 54, 71-210 Szczecin, Poland

**Keywords:** diet, sucrose, estrous cycle, sex hormone, estrogen, progesterone, gonadotropin

## Abstract

Relationships between diet, sex hormone concentrations, and the estrous cycle are important from the perspective of infertility and estrogen-dependent disease prevention and treatment. Four dietary interventions reflecting modern eating behaviors were explored. The study involved 50 female rats divided into five feeding groups. The impact of the amount of sucrose consumed (9% and 18% of the dietary energy content), alternating high-sucrose and sucrose-free diets, and a high-sucrose diet combined with intermittent one-day fasting on the estrous cycle and sex hormone concentrations in female rats was assessed. Even low amounts of dietary sucrose (9% of the dietary energy content) were found to lead to increased estradiol (E2) concentrations and decreased progesterone (Pg) concentrations. A high-sucrose diet, even when periodically applied, additionally led to a reduced concentration of luteinizing hormone (LH). The largest changes in the hormones tested were observed with one-day fasting coupled with the high-sucrose diet; in addition, the estrous phase was shortened and the estrous cycle was disrupted. The results of this study show that both the amount of dietary sucrose and also its uptake pattern affect the estrous cycle and sex hormone concentrations in female rats.

## 1. Introduction

Studies have demonstrated that a high-sugar diet applied to animal models can disturb their carbohydrate metabolism, induce insulin resistance, and cause lipid disorders, which are often associated with excessive weight gain and the accumulation of adipose tissue in the abdominal area [1,2,3,4,5,6]. As metabolic changes and reproductive health in females are closely connected and interact [7,8], disturbed carbohydrate–lipid metabolism may affect female reproductive health and reduce the chances of producing offspring [9,10]. It has been shown that fertility disorders are closely related to the increasing incidence of metabolic diseases (obesity, type 2 diabetes, and metabolic syndrome) caused by, among others, changes in eating patterns. It has been shown that contemporary eating habits, related to, inter alia, the excessive consumption of sugars, can change reproductive potential [11]. For example, in female rats, a high-sugar diet significantly lowered the relative weights of the ovary and uterus. It also significantly reduced the frequency of estrous and diestrus phases but caused significant increases in the metestrus phase of the estrous cycle and follicle-stimulating hormone levels in comparison with the control group [12]. It has also been shown that, in female rats, high-fat, high-sugar diets lead to abnormal estradiol, progesterone, and luteinizing hormone release before ovulation. The high-fat, high-sugar diet also resulted in altered basal levels of testosterone and luteinizing hormone [13]. Moreover, experimental studies have provided evidence that early exposure of female rats (21 days old) to a high-sugar diet leads to an increase in the number of cystic and atretic antral ovarian follicles, which were correlated with hypertrophy of periovarian adipocytes. Females on a high-sugar diet were found to enter puberty much earlier than controls [14]. However, research on rodents assessing the impact of sugar consumption on health, including reproductive health, usually focuses on high doses of sucrose (30–70% of sucrose in the energy value of the diet). There are no studies on the impact of various attempts to reduce sucrose intake in this respect. There are different methods used to reduce both the consumption of simple sugars and body weight, but their efficiency is rather low [15]. They involve, inter alia, a temporary elimination of sucrose from the diet, but the high-sugar diet is often returned to, alternating with the sugar-free diet. Attempts at reducing body weight also include intermittent one-day fasting, periodic application of restrictive diets, or a temporary adherence to the principles of proper nutrition, followed by a return to unhealthy eating habits [16]. Such behaviors may change lipid metabolism and intensify free-radical reactions [17] because the frequently changing diet composition may be perceived by the body as a stressor [18].

Our previous studies, in which we used sucrose doses in the amount of 10–18% of the energy value of the diet and made various attempts to reduce its consumption, showed that an increased dietary sucrose content, periodic fasting, or frequent changes in the diet composition (sugar-free vs. high-sugar diet) brought about adverse metabolic changes and increased free-radical reactions in the ovaries and uterus of female rats [19,20]. The demonstrated changes may modify the secretion of sex hormones by the gonads (estrogens and progesterone), also influencing, through a feedback loop, the secretion of luteinizing and follicle-stimulating hormones, and thus the course of the sexual cycle [21]. The obtained results allow for conclusions regarding the relationship between the consumption of sucrose and the concentration of sex hormones and the course of the estrous cycle, which may affect the reproductive health and fertility of female rats, and, through the influence of estrogens, modulate the possibility of developing estrogen-dependent neoplasms [22].

In this study, we aimed to find out if and how diets with different sucrose contents, i.e., (i) low-sucrose and (ii) high-sucrose contents, as well as dietary interventions intended to limit sucrose consumption, i.e., (iii) alternating high-sucrose and sucrose-free diets, and (iv) an intermittent one-day release from the high-sucrose diet, affect the estrous cycle and the sex hormone concentrations in female rats. Rat models are commonly used in reproductive function research possibly due to their well-characterized, short and precise length of estrous cycle and secure handling. Studying the estrous cycle in experimental animals is a useful measure of the integrity of the hypothalamic–pituitary–ovarian axis, determining the fertility of animals, and the functioning of reproductive status of the female reproductive system [23,24,25]. Therefore, the obtained results may contribute to explaining the causes of disorders in the reproductive health of females.

## 2. Materials and Methods

### 2.1. Animals and Study Design

A total of 50 female Wistar rats (Department of Toxicology of Poznań University of Medical Sciences) were single-housed under a 12/12 h light/dark cycle at room temperature (21 ± 2 °C) with a relative humidity of 55–60%. The rats were 3 months old, which, according to the literature data [26], are animals that have reached sexual maturity, with an initial body weight of 204.7 ± 15.1 g and final body weight 231.3 ± 17.0. After a week of adaptation, the animals were divided into five groups (*n* = 10 each) of equal body weight. The research was approved by the Local Research Ethics Committee (Approval No. 18/2015) in accordance with the European Convention for the Protection of Vertebrate Animals used for Experimental and other Scientific Purposes, Council of Europe, Strasbourg 1986.

The composition of the basic feed (BF) was in line with the nutrient requirements according to AIN-93M [27]. The BF contained mainly whole wheat and maize grain, which, in the modified feeds (MF1 and MF2), were partly substituted with wheat flour (type 500) and sucrose. In MF1, sucrose accounted for 8% of the component composition, and the percentage of energy derived from sucrose was 9.1%. In MF2, sucrose accounted for 16% of the component composition, and the percentage of energy derived from sucrose was 18%.

Because animal models are widely used to study the effects of dietary components, including simple sugars, on metabolism, such a composition of the diets and the nutrition scheme used allows one to reflect the changes in nutrition taking place at present. The sugar amount was set, taking into account the WHO recommendations (MF1) and the composition of modern diets of many people in which the percentage of added sugars is up to 20% (MF2) [28,29]. Table S1 shows the detailed composition of the feeds used in the experiment.

The chemical composition of the feeds was determined in accordance with the methodology provided by AOAC [30]. In the collected feeds samples, we examined the content of total nitrogen converted into an amount of protein (Kjeldahl method, on a Kjeltec 2100 system from Foss Tecator, Hilleroed, Denmark), crude fat (Soxhlet method, on a Soxtec HT6 system from Foss Tecator, Hilleroed Denmark), crude fiber (gravimetric method, on an ANKOM 220 Fiber Analyzer, ANKOM Technology, Macedon, NY, USA), and dry matter and ash (gravimetric method, on an SUP-4M laboratory dryer Wawa-Med, Warsaw, Poland and a muffle furnace Czylok, Jastrzębie Zdrój, Poland, respectively). The difference between the dry mass and the sum of the remaining solid components allowed us to estimate the carbohydrate content. The metabolic energy content was estimated using the following conversion factors: for protein and carbohydrates, 17 kJ/g (4.0 kcal/g), and for fats, 37 kJ/g (9.0 kcal/g) [31]. The results of the analyses and calculations are presented in Table S2.

Animal nutrition during the experiment:

(i) The CG (control group) was fed BF;
(ii) The SBG (sugar-balanced group) received MF1 containing 9.1% of the energy value from sucrose over the whole experimental period;
(iii) The HSG (high-sugar group) received MF2 containing 18% of the energy value from sucrose;
(iv) The AFG (alternately fed group) received BF and MF2 alternately every second week (in even weeks, BF, and in odd weeks, MF2);
(v) The PSG (periodically starved group) received MF2 and were starved one day in every week (6 days on MF2 + 1 day starvation).

The consumption of sucrose in the SBG and AFG during the entire experimental period (8 weeks) was similar and amounted to approx. 9% of the energy value of the diet. The consumption of sucrose in the HSG and PSG during the entire experimental period was similar and amounted to approx. 18% of the energy value of the diet.

The structure of the experiment allowed us to determine the effect of the sucrose intake (CG vs. SBG vs. HSG); the scheme of sucrose administration, i.e., small amounts constantly or larger amounts alternately with a sucrose-free diet (SBG vs. AFG); and the use of one-day starvation in combination with a diet containing 18% of the energy value from sucrose (HSG vs. PSG) on the tested parameters. Water and solid food were available ad libitum.

The body weight of the animals was determined at the beginning and end of the experiment. Over the 8 weeks of the experiment, the amount of food intake was measured daily. From the 27th day of the experiment for 28 days every morning from 8 a.m. to 9 a.m., the phase of the estrous cycle was determined. According to the methodology described by Marcondes et al. [32], by examining the type and number of cells present in the vaginal lavage, the estrous phase was determined. Vaginal secretions were collected with a plastic pipette filled with 10 mL of saline (NaCl 0.9%) by inserting the tip into the rat vagina. Vaginal fluid was placed on glass slides. Unstained material was immediately observed under a light microscope with 10 and 40× objective lenses. Three types of cells were recognized: epithelial cells, which are round and nucleated; cornified cells, which are irregular without a nucleus; and leukocytes, which are slightly rounded. Their relative proportions were used for the determination of the estrous cycle phases. As in the initial period, sampling could be a stress for the animals that may have influenced the phase of the cycle; thus, the measurement from the first 7 days was not included in the analyses. During this period of 21 days, the total number of days in the estrous phase, in which ovulation occurs and females are fertile, was calculated. After the eighth week of treatment, the assigned rats, after determining that they were in the estrous phase, were fasted (12 h), and anesthetized with an intramuscular injection (10 mg/kg b.w.) of Ketanest (Pfizer Ireland Pharmaceuticals, Ringaskiddy, Ireland).

### 2.2. Sample Collection

Plasma for the analyses was obtained by centrifugation (1000× *g*, 10 min, 4 °C) of the blood collected from the heart into vacuum tubes (Sarstedt, Nümbrecht, Germany). EDTA was used as an anticoagulant. The obtained plasma was deep-frozen (−70 °C) until analysis (maximum one month).

### 2.3. Biochemical Analysis

Plasma luteinizing hormone (LH, Rat ELISA kit Fine Test Wuhan Fine Biotech Co., Wuhan, China, Cat. No. ER1123), follicle-stimulating hormone (FSH, Rat ELISA kit Fine Test Wuhan Fine Biotech Co., Wuhan, China, Cat. No. ER0960), estradiol (E2, Rat ELISA kit Fine Test Wuhan Fine Biotech Co., Wuhan, China, Cat. No. ER1507), progesterone (Pg, Rat ELISA kit Fine Test Wuhan Fine Biotech Co., Wuhan, China, Cat. No. ER1255), testosterone (T, Rat ELISA kit Fine Test Wuhan Fine Biotech Co., Wuhan, China, Cat. No. ER1462), and sex hormone-binding globulin (SHBG, Rat ELISA kit Fine Test Wuhan Fine Biotech Co., Wuhan, China, Cat. No. ER0290) concentrations were assayed using a monoclonal antibody against rat hormones according to the manufacturer’s instructions on an Epoch spectrophotometer (BioTek Instruments, Winooski, VT, USA). The coefficients of variation (CV) of the intra- and inter-assay for the tests were, respectively, 8% and 10% for the E2, LH, FSH, Pg, T, and SHBG assay kits.

### 2.4. Statistical Analysis

Comparisons between groups were performed using one-way ANOVA and the Tukey test in the Statistica 12.0^®^ program (Statsoft, Tulsa, OK, USA). The homogeneity of variance and normality of distribution were assessed using Levene’s test and the modified Shapiro–Wilk test. When the data were not normally distributed and/or variances were not equal, logarithmic transformation was performed. All the numerical data were expressed as means ± S.E.M. Significance was established at *p* < 0.05.

## 3. Results

Table 1 shows the total energy and sucrose consumption of the test animals, as well as the per cent contribution of their energy derived from dietary sucrose. The results demonstrated that the total dietary energy supply in the PSG was significantly higher than that in the CG and AFG.

The sucrose consumption in the HSG and PSG was comparable and amounted to about 52.2 g/100 g b.w., which accounted for 18% of the total dietary energy. The sucrose consumption in the SBG and AFG groups was also similar as (about 26 g/100 g b.w., i.e., 9.1% of the total dietary energy).

### 3.1. The Estrous Cycle

Table 2 summarizes the data on the estrous cycle of the females tested; details of the estrous cycles are given in Tables S3–S7.

The duration of the individual estrous phases did not differ significantly between the SBG and CG. However, the HSG, AFG, and PSG showed a significantly shorter duration of the phases as compared to the control (CG), with the PSG also differing significantly from the SBG (Table 2).

All the females in the CG and SBG showed a regular sexual cycle of a normal length. In contrast, the HSG, AFG, and PSG included females whose cycles were irregular and of abnormal duration. The highest number of females (7 out of 10) with irregular cycles was found in the PSG (Table 2).

### 3.2. Hormone Concentrations

The blood serum concentrations of sex hormones and sex hormone-binding globulin in the females tested are shown in Table 1.

**Figure 1:**
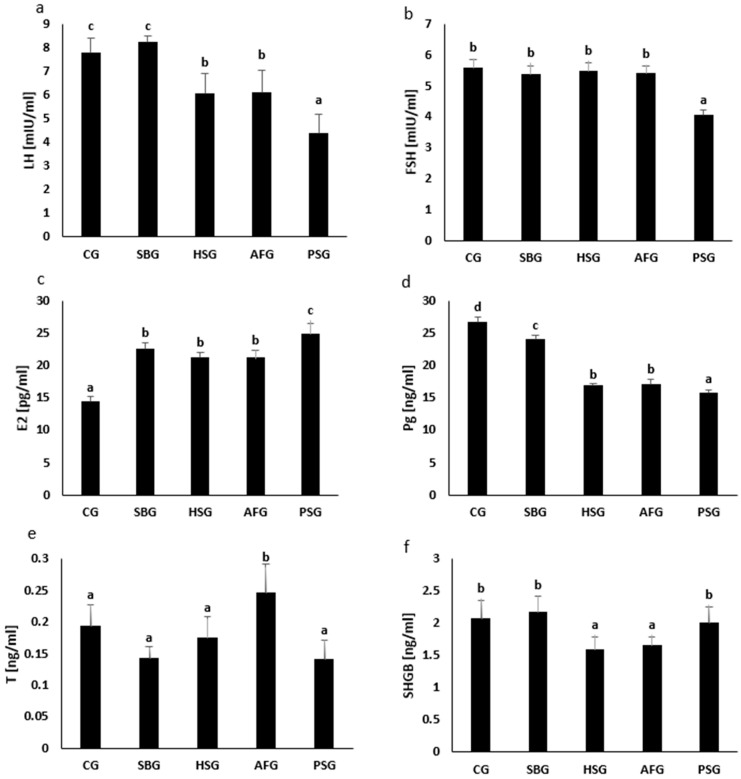
Effects of various sucrose-containing diets and feeding patterns on the serum concentrations of (**a**) luteinizing hormone (LH), (**b**) follicle-stimulating hormone (FSH), (**c**) estradiol (E2), (**d**) progesterone (Pg), (**e**) testosterone (T), and (**f**) sex hormone-binding globulin (SGBG) in female rats. CG, control group; SBG, sugar-balanced group; HSG, high-sugar group; AFG, alternately feeding group; PSG, periodically starved group; a, b, c, and d, bars marked with different letters reflect significantly different (*p* ≤ 0.05) outcomes; Tukey test, *p*-values are given in Table S8; *n* = 50 (10 per each group).

The analysis of the changes in the concentration of gonadotropins (luteinizing hormone, LH; and follicle-stimulating hormone, FSH) in the groups of the tested animals showed that the concentration of LH in the SBG group (8.21 ± 0.52 mIU/mL) did not change significantly compared to that in the CG (7.77 ± 1.24 mIU/mL). However, in the HSG (6.02 ± 1.72 mIU/mL), AFG (6.09 ± 1.87 mIU/mL), and PSG (4.38 ± 1.59 mIU/mL) groups, we found a significant decrease in LH concentration as compared to the CG. Moreover, we found that in the PSG group, the LH concentration was significantly lower, not only compared to that in the CG but also to that in the SBG, HSG, and AFG (Figure 1a, Table S8).

We did not find any significant changes in FSH concentration in the SBG (5.37 ± 0.56 mIU/mL), HSG (5.48 ± 0.55 mIU/mL), or AFG (5.39 ± 0.52 mIU/mL) groups as compared to the CG (5.56 ± 0.55 mIU/mL) group. However, we found that in the PSG group (4.05 ± 0.35 mIU/mL), the FSH concentration was significantly lower than that in the CG, SBG, HSG, and AFG groups (Figure 1b, Table S8).

Regarding the analysis of changes in the concentration of steroid hormones (estradiol, E2; progesterone, Pg; testosterone, T), we found a significant increase in E2 concentration in each study group (SBG, 22.5 ± 1.95 pg/mL; HSG, 21.1 ± 1.65 pg/mL; AFG, 21.1 ± 2.52 pg/mL; PSG, 24.8 ± 3.52 pg/mL) in relation to CG (14.3 ± 1.84 pg/mL). Moreover, we found that in the PSG group, the E2 concentration was significantly higher not only compared to the CG but also to the SBG, HSG, and AFG (Figure 1c, Table S8). There was a significant decrease in Pg concentration in each study group (SBG, 24.0 ± 1.39 ng/mL; HSG, 16.9 ± 0.46 ng/mL; AFG, 17.1 ± 1.41 ng/mL; PSG, 15.7 ± 1.17 ng/mL) in relation to the CG (26.7 ± 1.60 ng/mL). Moreover, in the HSG and AFG groups, the Pg concentration was significantly lower not only compared to the CG but also to the SBG, and in the PSG, it was lower also in comparison to the SBG, HSG, and AFG (Figure 1d, Table S8). There was a significant increase in T concentration in the AFG (0.24 ± 0.09 ng/mL) compared to the CG (0.19 ± 0.06 ng/mL), SBG (0.14 ± 0.03 ng/mL), HSG (0.17 ± 0.07 ng/mL), and PSG (0.14 ± 0.06 ng/mL (Figure 1e, Table S8).

We found no significant changes in the concentration of sex hormone binding globulin (SHBG) in the SBG (2.17 ± 0.47 ng/mL) and PSG (1.99 ± 0.50 ng/mL) as compared to the CG (2.06 ± 0.56 ng/mL). However, we found that the concentration of SHGB in the HSG (1.58 ± 0.40 ng/mL) and AFG (1.65 ± 0.27 ng/mL) was significantly lower in comparison to the CG and the SBG and PSG (Figure 1f, Table S8).

## 4. Discussion

This study assessed the effects of the amount of dietary sucrose (sucrose balanced diet group, SBG; high-sucrose diet group, HSG) and the attempts to limit its consumption (alternating high-sucrose and sucrose-free diets, AFG, and a high-sucrose diet combined with intermittent one-day fasting, PSG) on the estrous cycle and the concentration of sex hormones in female rats.

The results showed that the sucrose-containing diets, regardless of the sucrose level (SBG and HSG), and the attempts to restrict sucrose consumption (AFG and PSG) significantly increased the E2 concentration in the animal blood, with the Pg concentration becoming lower.

Moreover, the high-sucrose diet in the HSG, AFG, and PSG significantly lowered the peripheral LH levels, even when increased amounts of sucrose were consumed only intermittently (AFG) or when sucrose consumption was interrupted by fasting (PSG). The T concentration in the AFG increased, while that of the FSH decreased, with estrous cycle disorders being the most frequent in the PSG.

Throughout most of the estrous cycle, gonadotropin-releasing hormone (GnRH) and LH/FSH secretion are controlled by the negative estrogen feedback [33,34,35]. In our study, the high-sucrose diets (HSG, AFG, and PSG) significantly decreased the LH concentration in the estrous phase, which could have been caused by negative feedback related to the increased E2 concentration at the time [36]. Estrogen has been demonstrated to induce a GnRH/LH surge in female rodents through kisspeptin neurons (Kiss1); high estrogen levels have been shown to inhibit Kiss1 gene expression, and thus the LH surge [37,38]. Kiss1 expression increases after ovariectomy and is suppressed when E2 is administered to the animals. These changes are also reflected in changing blood LH levels [39]. Moreover, a reduced Kiss1 gene expression has been revealed in obese mice [40], with a fat- and sugar-rich diet being associated with a decreased LH surge at high E2 levels [13]. Therefore, the results obtained in this study may be explained by chronic exposure to an increased amount of dietary sucrose leading to an increased E2 concentration and thus downregulation of Kiss1 expression, which reduces the basal LH levels. Such a mechanism is possible with long-term exposure to high dietary sucrose contents (HSG, AFG, and PSG), but does not operate with exposure to a low sucrose content (SBG).

That high-sucrose diets reduce LH levels suggests that the effect may be due to, inter alia, factors related to the profile of metabolic disorders that accompany sucrose consumption. We have already shown [19,20] that a high-sucrose diet (HSG and PSG), even when administered periodically (AFG), significantly increases glycemia, the HOMA-IR index, and LDL-cholesterol, and reduces the HDL-cholesterol concentration as compared to the group fed a low-sucrose diet (SBG).

Therefore, it seems that, regardless of the total amount of sucrose in the diet and the amount of sucrose-derived energy (SBG vs. AFG), excess sucrose consumption, even if it is periodic or alternates with fasting, results in dyslipidemia and insulin resistance. Consequently, our current finding of reduced LH concentrations in the HSG, AFG, and PSG may be related to the influence of sucrose on insulin signaling.

The correct insulin signaling in the central nervous system is known at present to be essential for appropriate activation of the gonadotropic axis by influencing GnRH and LH release [41]. Low-circulating insulin levels in diabetic rats have been shown to reduce both GnRH release from the hypothalamus and the LH-releasing pituitary cell response to GnRH [42]; it has also been demonstrated that central insulin administration directly stimulates GnRH neurons, resulting in pulsatile LH release [43]. A potential importance of insulin under these conditions is additionally emphasized by the observation that diabetes mellitus is accompanied by reproductive disorders resulting from impaired LH secretion in rodents [44]. Moreover, insulin resistance is strongly associated with lower levels of HDL-cholesterol, which modulates glucose metabolism by activating AMP-dependent protein kinase (AMPK) [45]. Hence, both insulin resistance and the low HDL-cholesterol levels shown in our previous studies in the HSG, AFG, and PSG [19,20] may have contributed to the disruption of the hypothalamic–pituitary axis, including the reduction in LH synthesis and/or secretion [46]. Insulin is a signal that links metabolism with reproduction, particularly since Kiss1 neurons are sensitive to circulating metabolic signals such as leptin and insulin, which confirms their importance in mediating the body’s response to nutrition or malnutrition [47]. Moreover, insulin may exert an effect on the frequency of pulsatile GnRH release, which determines the proportion of LH and FSH synthesis and secretion. Low-frequency GnRH pulsations stimulate FSH secretion, with LH secretion being stimulated by the high-frequency pulsations [47]. This allows us to conclude that the low LH levels in the HSG and AFG could be related to the reduced GnRH pulsation which was, nevertheless, sufficient for appropriate FSH secretion.

We also found that the cycle of one-day fasting coupled with the high-sucrose diet (PSG) inhibited gonadotropin secretion much more than the high-sucrose diet (HSG) did, despite a similar uptake of sucrose and energy. The PSG females showed LH and FSH concentrations significantly lower than those in the CG as well as in the HSG and AFG. The PSG rats also showed the largest changes in the E2 and Pg concentrations. Such changes in the sex hormone concentrations in the PSG could be the cause, but also as a consequence of the estrous cycle dysregulation, found in 7 out of 10 the PSG females. Studies on the effects of feeding restrictions on gonadotropin secretion in female rats showed that a 24 h fast every other day for 12 weeks led to reduced serum LH levels [48]. Gonadotrophin levels were monitored during a 72 h fast. Under these conditions, the Kiss1 mRNA level in the hypothalamus was observed to drop, which was accompanied by inhibited GnRH secretion and significantly reduced basal serum LH and FSH levels in the fasting females. In addition, central injections of kisspeptin have been shown to reverse the fasting-induced inhibition of GnRH secretion [49]. Thus, the pattern of gonadotropin secretion suppression, i.e., the reduced LH and FSH levels, in the PSG is consistent with the findings of previous studies on the effects of feeding restrictions on gonadotropin secretion. The largest increase in the E2 concentration and the sharpest drop in LH and Pg, as well as the FSH concentration, in the PSG indicates that intermittent fasting is an important factor that adversely modifies sex hormone secretion.

The Pg concentration was observed to decrease in the animals fed a high-sugar diet (HSG, AFG, and PSG). Reductions in the Pg concentration in mice on a high-sugar diet have also been reported by Saben et al. [50]. Their study demonstrated that high fructose consumption for 6 weeks significantly lowered the Pg levels in the female blood serum. After mating, the decreased Pg production and impaired decidualization of endometrial stromal cells led to increased pregnancy loss. In contrast, Tobiansky et al. [51] reported in their study on rodents that a sucrose-containing diet increased the Pg concentration in the blood serum and brain of rats. However, those authors looked at effects of long-term sucrose consumption before and after pregnancy and childbirth, two physiological states associated with severe changes in the hormone balance, which may explain the differences with respect to our results.

Appropriate Pg production and release is a result of a normal pre-ovulatory LH surge, which brings about luteinization of granulosal and thecal cells and alters the steroidogenic pathway so that Pg is the major steroid hormone secreted [52]. The lower blood Pg concentration in the animals fed high-sucrose diets (HSG, AFG, and PSG) may be a consequence of a reduced LH concentration. Sagae et al. [53], who fed rats cafeteria-style diets, showed a decreased pre-heat LH surge along with reduced Pg levels. The cause for the lower Pg concentrations in HSG, AFG, and PSG could also be due to the low HDL-cholesterol concentration [19,20], which may negatively affect Pg synthesis. In rodents, cholesterol derived from HDL lipoproteins and intercepted from the circulation is the Pg synthesis precursor molecule [54]. However, this does not explain the low Pg concentration in the SBG, which showed normal LH levels and no reduction in HDL-cholesterol levels.

The data from the HSG, AFG, and PSG might be explained by reference to our earlier studies [19,20] demonstrating that a high-sucrose diet led to oxidative stress and weakened antioxidant defense in the reproductive tissues (uterus and ovary) of female rats. Oxidative stress plays an important role in lutein granulosa deficiency [55], which explains the low Pg levels in mice experiencing oxidative stress [21,56]. Reduced Pg levels have been also shown to decrease superoxide dismutase expression and thus the generation of free radicals in the uterus, which ultimately leads to endometrial exfoliation and embryo implantation failure [57]. However, the low Pg concentration in the SBG still remains unclear.

From the perspective of infertility prevention and/or treatment, the relationships between the diet, estrogen, progesterone levels, the course of the estrous cycle are important. They are important also in the context of estrogen-related disease prevention because increased estrogen levels and reduced progesterone levels increase the risk of breast cancer [22].

Reduced food consumption may lead to weight loss [58], or, as in our study, to maintaining a stable body weight. However, caloric restrictions, even when periodically applied, greatly affect sex hormone secretion and contribute to estrous cycle disruptions in female rats. However, further research is needed to elucidate the mechanism of these effects. Involvement of metabolic signals, which modulate the relationship between the energetic state and the hypothalamic–pituitary–gonadal axis hormone secretion, is suggested. Involvement of, inter alia, leptin, ghrelin, neuropeptide Y, adiponectin, cortisol, insulin, and IGF-1 has been considered [59] but remains beyond the scope of this study.

Limitation of the study. A limitation of the analyses, which was limited to the determination of sex hormone levels, without GnRH levels or kisspeptin expression, and their performance in rodents whose sexual cycle is similar to that observed in humans but differs significantly in the length and duration of individual phases. Another limitation was that we determined the sexual cycle phase only once a day.

## 5. Conclusions

The data obtained in this study show that both the presence of dietary sucrose and as its quantity and supply patterns, associated with attempts to reduce its consumption, affect sex hormone concentrations and the estrous cycle in female rats. Even low (up to 10% of the dietary energy content) amounts of dietary sucrose increased the E2 concentration and decreased the Pg concentration. A diet with 18% of the energy supplied by sucrose, even when applied intermittently, resulted in a reduced concentration of LH and SHBG. Intermittent fasting coupled with a high-sucrose diet produced the most pronounced changes in the concentrations of the hormones tested (the lowest concentration of LH, FSH, and Pg and the highest concentration of E2), shortened the estrous phase, and disrupted the estrous cycle in 7 out of 10 females.

Rat models are commonly used for endocrinological research [57], such as assessing the effects of various diets [13,53], dietary components [60] or intermittent fasting-dietary restriction [48] on the levels of hypothalamic–pituitary–ovarian axis hormones. Although the results of our study showed that both the amount of dietary sucrose and also its uptake pattern affect the estrous cycle and sex hormone concentrations in female rats: (i) we did not assess the fertility parameters in the research; therefore, it is difficult to say whether and how the changes we observe will translate into fertility and reproduction, and (ii) rodent physiology differs from human physiology in many aspects [61]; therefore, the results obtained in our research should be extrapolated to humans with great caution.

## Figures and Tables

**Table 1:** Body weights, feed, energy, and sucrose consumption of the tested animals.

Trait	CG	SBG	HSG	AFG	PSG
Initial b.w. (g)	205 ± 17.8	205 ± 16.0	204 ± 13.0	205 ± 15.8	205 ± 16.0
Final b.w. (g)	230 ± 19.8	232 ± 17.8	231 ± 14.1	233 ± 16.8	230 ± 16.7
Feed intake (g/100 g b.w.)	335 ± 8.5	328 ± 13.8	326 ± 8.3	330 ± 6.9 (169 BF + 161 MF1)	333 ± 10.2
Energy intake (kcal/100 g b.w.)	1142 ± 28.5 ^a^	1154 ± 45.3 ^a,b^	1157 ± 27.1 ^a,b^	1147 ± 24.6 ^a^	1181 ± 33.2 ^b^
Sucrose intake (g/100 g b.w.)	0 ^a^	26.2 ± 1.1 ^b^	52.2 ± 1.3 ^c^	25.8 ± 1.0 ^b^	52.3 ± 1.6 ^c^
Contribution of sucrose-derived energy to the total dietary energy content (%)	0	9.1%	18%	9%	18%

CG, control group; SBG, sugar-balanced group; HSG, high-sugar group; AFG, alternately fed group; PSG, periodically starved group; BF, basic feed; MF1, modified feed 1; b.w., body weight; ^a,b,c^, different letters in the same line are statistically different, *p* ≤ 0.05; *n* = 50 (10 per each group).

**Table 2:** Summary of the estrous cycles in the females studied.

Trait	CG	SBG	HSG	AFG	PSG
Duration of the estrous phase (days)	7.7 ± 1.25 ^c^	6.6 ± 0.84 ^b,c^	5.6 ± 0.7 ^a,b^	5.7 ± 1.16 ^a,b^	4.6 ± 1.07 ^a^
Females with irregular cycles (number)	0/10	0/10	3/10	2/10	7/10

CG, control group; SBG, sugar-balanced group; HSG, high-sugar group; AFG, alternately fed group; PSG, periodically starved group; ^a,b,c^, different letters in the same line are statistically different, *p* ≤ 0.05; *n* = 50 (10 per each group).

## Data Availability

Data available from the authors of the study.

## Supplementary Materials

The following supporting information can be downloaded at: https://www.mdpi.com/article/10.3390/nu14204350/s1, Table S1: Component composition of diets; Table S2: Chemical composition of diets; Table S3: The course of the estrous cycle in females from the control group (CG); Table S4: The course of the estrous cycle in females from the sugar balanced group (SBG); Table S5: The course of the estrous cycle in females from the high sugar group (HSG); Table S6. The course of the estrous cycle in females from the alternately fed group (AFG); Table S7: The course of the estrous cycle in females from the periodically starved group (PSG). Table S8: *p*-values for the statistics of hormone concentration determinations.

## References

[1] Villegas-Romero M., Castrejón-Téllez V., Pérez-Torres I., Rubio-Ruiz M.E., Carreón-Torres E., Díaz-Díaz E., Del Valle-Mondragón L., Guarner-Lans V. (2018). Short-Term Exposure to High Sucrose Levels near Weaning Has a Similar Long-Lasting Effect on Hypertension as a Long-Term Exposure in Rats. Nutrients.

[2] Cao L., Liu X., Cao H., Lv Q., Tong N. (2012). Modified high-sucrose diet-induced abdominally obese and normal-weight rats developed high plasma free fatty acid and insulin resistance. Oxid. Med. Cell. Longev..

[3] Sun S., Hanzawa F., Umeki M., Ikeda S., Mochizuki S., Oda H. (2018). Time-restricted feeding suppresses excess sucrose-induced plasma and liver lipid accumulation in rats. PLoS ONE.

[4] Sadowska J., Bruszkowska M. (2019). Assessing the effect of sugar type and form of its intake on selected parameters of carbohydrate-lipid metabolism and plasma atherogenic indices in rats. Rocz. Panstw. Zakl. Hig..

[5] Melo B.F., Sacramento J.F., Ribeiro M.J., Prego C.S., Correia M.C., Coelho J.C., Cunha-Guimaraes J.P., Rodrigues T., Martins I.B., Guarino M.P. (2019). Evaluating the Impact of Different Hypercaloric Diets on Weight Gain, Insulin Resistance, Glucose Intolerance, and its Comorbidities in Rats. Nutrients.

[6] Rodríguez-Correa E., González-Pérez I., Clavel-Pérez I., Contreras-Vargas Y., Carvajal K. (2020). Biochemical and nutritional overview of diet-induced metabolic syndrome models in rats: What is the best choice?. Nutr. Diabetes.

[7] Della Torre S., Benedusi V., Fontana R., Maggi A. (2014). Energy metabolism and fertility: A balance preserved for female health. Nat. Rev. Endocrinol..

[8] Nikanfar S., Oghbaei H., Rastgar Rezaei Y., Zarezadeh R., Jafari-Gharabaghlou D., Nejabati H.R., Bahrami Z., Bleisinger N., Samadi N., Fattahi A. (2021). Role of adipokines in the ovarian function: Oogenesis and steroidogenesis. J. Steroid. Biochem. Mol. Biol..

[9] Christiansen J.J., Djurhuus C.B., Gravholt C.H., Iversen P., Christiansen J.S., Schmitz O., Weeke J., Jørgensen J.O., Møller N. (2007). Effects of cortisol on carbohydrate, lipid, and protein metabolism: Studies of acute cortisol withdrawal in adrenocortical failure. J. Clin. Endocrinol. Metab..

[10] Laugero K.D., Falcon L.M., Tucker K.L. (2011). Relationship between perceived stress and dietary and activity patterns in older adults participating in the Boston Puerto Rican Health Study. Appetite.

[11] Panth N., Gavarkovs A., Tamez M., Mattei J. (2018). The influence of diet on fertility and the implications for public health nutrition in the United States. Front. Public Health.

[12] Eunice O., Homahinuchu J.H.H., Ariyo J.D. (2021). Sugar intake disrupts some reproductive functions in female wistar rats. J. Infertil. Reprod. Biol..

[13] Volk K., Pogrebna V.V., Roberts J.A., Zachry J.E., Blythe S.N., Toporikova N. (2017). High-fat, high-sugar diet disrupts the preovulatory hormone surge and induces cystic ovaries in cycling female rats. J. Endocr. Soc..

[14] De Melo G.B., Soares J.F., Costa T., Benevides R., Vale C.C., Paes A., Gaspar R.S. (2021). Early Exposure to High-Sucrose Diet Leads to Deteriorated Ovarian Health. Front. Endocrinol..

[15] Di Monaco R., Miele N.A., Cabisidan E.K., Cavella S. (2018). Strategies to reduce sugars in food. Curr. Opin. Food Sci..

[16] Larson N.I., Neumark-Sztainer D., Story M. (2009). Weight control behaviors and dietary intake among adolescents and young adults: Longitudinal findings from Project EAT. J. Am. Diet. Assoc..

[17] Niño O.M.S., da Costa C.S., Torres K.M., Zanol J.F., Freitas-Lima L.C., Miranda-Alves L., Graceli J.B. (2020). High-refined carbohydrate diet leads to polycystic ovary syndrome-like features and reduced ovarian reserve in female rats. Toxicol. Lett..

[18] Nwogueze B.C., Ojieh A.E., Wilson J.I., Ovuakporaye S.I., Ohwin P.E., Aisuodionoe E.M., Daubry T., Agbonifo-Chijiokwu E., Eke C.N., Omeru O. (2021). Down regulatory response of reproductive potentials in stress-induced rats supplemented with clomifene citrate: The fate of infertility. Biomed. Pharmacother..

[19] Sadowska J., Dudzinska W., Skotnicka E., Sielatycka K., Daniel I. (2019). The impact of a diet containing sucrose and systematically repeated starvation on the oxidative status of the uterus and ovary of rats. Nutrients.

[20] Sadowska J., Dudzinska W., Dziaduch I. (2021). Effects of different models of sucrose intake on the oxidative status of the uterus and ovary of rats. PLoS ONE.

[21] Shi L., Zhang J., Lai Z., Tian Y., Fang L., Wu M., Xiong J., Qin X., Luo A., Wang S. (2016). Long-Term Moderate Oxidative Stress Decreased Ovarian Reproductive Function by Reducing Follicle Quality and Progesterone Production. PLoS ONE.

[22] Shull J.D., Dennison K.L., Check A.C., Trentham-Dietz A. (2018). Rat models of 17β-estradiol-induced mammary cancer reveal novel insights into breast cancer etiology and prevention. Physiol. Genom..

[23] Andrews W.W., Ojeda S.R. (1981). A detailed analysis of the serum luteinizing hormone secretory profile in conscious, free-moving female rats during the time of puberty. Endocrinology.

[24] Auta T., Hassan A.T. (2016). Alteration in oestrus cycle and implantation in *Mus musculus* administered aqueous wood ash extract of *Azadirachta indica* (neem). Asian Pacific J. Reprod..

[25] Ajayi A.F., Akhigbe R.E. (2020). Staging of the estrous cycle and induction of estrus in experimental rodents: An update. Fertil. Res. Pract..

[26] Ghasemi A., Jeddi S., Kashfi K. (2021). The laboratory rat: Age and body weight matter. EXCLI J..

[27] Reeves P.G., Nielsen F.H., Fahey G.C. (1993). AIN-93 purified diets for laboratory rodents: Final report of the American Institute of Nutrition ad hoc writing committee on the reformulation of the AIN-76 rodent diet. J. Nutr..

[28] Yang Q., Zhang Z., Gregg E.W., Flanders W.D., Merritt R., Hu F.B. (2014). Added sugar intake and cardiovascular diseases mortality among US adults. JAMA Intern. Med..

[29] World Health Organization (2015). WHO Guideline: Sugars Intake for Adults and Children.

[30] AOAC, Association of Official Analytical and Chemists (2016). Official Methods of Analysis.

[31] FAO (2003). Chapter 2: Methods of Food Analysis. Food Energy—Methods of Analysis and Conversion Factors.

[32] Marcondes F.K., Bianchi F.J., Tanno A.P. (2002). Determination of the estrous cycle phases of rats: Some helpful considerations. Braz. J. Biol..

[33] McQuillan H.J., Han S.Y., Cheong I., Herbison A.E. (2019). GnRH pulse generator activity across the estrous cycle of female mice. Endocrinology.

[34] Smith J.T., Popa S.M., Clifton D.K., Hoffman G.E., Steiner R.A. (2006). Kiss1 neurons in the forebrain as central processors for generating the preovulatory luteinizing hormone surge. J. Neurosci..

[35] Shaw N.D., Srouji S.S., Histed S.N., Hall J.E. (2011). Differential effects of aging on estrogen negative and positive feedback. Am. J. Physiol. Endocrinol. Metab..

[36] Hall J., Strauss J., Barbieri R. (2004). Chapter 7—Neuroendocrine Control of the Menstrual Cycle. Yen and Jaffe’s Reproductive Endocrinology: Physiology, Pathophysiology, and Clinical Management.

[37] Kauffman A.S., Clifton D.K., Steiner R.A. (2007). Emerging ideas about kisspeptin–GPR54 signaling in the neuroendocrine regulation of reproduction. Trends. Neurosci..

[38] Roa J., Aguilar E., Diéguez C., Pinilla L. (2008). New frontiers in kisspeptin/GPR54 physiology as fundamental gatekeepers of reproductive function. Front. Neuroendocrinol..

[39] Minabe S., Uenoyama Y., Tsukamura H., Maeda K. (2011). Analysis of pulsatile and surge-like luteinizing hormone secretion with frequent blood sampling in female mice. J. Reprod. Develop..

[40] Quennell J.H., Howell C.S., Roa J., Augustine R.A., Grattan D.R., Anderson G.M. (2011). Leptin deficiency and diet-induced obesity reduce hypothalamic kisspeptin expression in mice. Endocrinology.

[41] Sliwowska J.H., Fergani C., Gawełek M., Skowronska B., Fichna P., Lehman M. (2014). Insulin: Its role in the central control of reproduction. Physiol. Behav..

[42] Dong Q., Lazarus R.M., Wong L.S., Vellios M., Handelsman D.J. (1991). Pulsatile LH secretion in streptozotocin-induced diabetes in the rat. J. Endocrinol..

[43] Kotani M., Detheux M., Vandenbogaerde A., Communi D., Vanderwinden J.M., Le Poul E., Brezillon S., Tyldesley R., Suarez-Huerta N., Vandeput F. (2002). Effect of centrally administered insulin on gonadotropin-releasing hormone neuron activity and luteinizing hormone surge in the diabetic female rat. Neuroendocrinology.

[44] Lee J., Lee H.C., Kim S.Y., Cho G.J., Woodruff T.K. (2019). Poorly-controlled Type 1 diabetes mellitus impairs LH-LHCGR signaling in the ovaries and decreases female fertility in mice. Yonsei Med. J..

[45] Chen M.J., Yang W.S., Hsiao C.K., Yang Y.S., Ho H.N. (2006). Low sex hormone-binding globulin is associated with low high-density lipoprotein cholesterol and metabolic syndrome in women with PCOS. Hum. Reprod..

[46] Chosich J., Bradford A.P., Allshouse A.A., Reusch E.B., Santoro N., Schauer I.E. (2017). Acute recapitulation of the hyperinsulinemia and hyperlipidemia characteristic of metabolic syndrome suppresses gonadotropins. Obesity.

[47] Qiu X., Dao H., Wang M., Heston A., Garcia L.D., Sangal A., Dowling A.R., Faulkner L.D., Molitor S.C., Elias C.F. (2015). Insulin and leptin signaling interact in the mouse kiss1 neuron during the peripubertal period. PLoS ONE.

[48] Kumar S., Kaur G. (2013). Intermittent fasting dietary restriction regimen negatively influences reproduction in young rats: A study of hypothalamo-hypophysial-gonadal axis. PLoS ONE.

[49] Castellano J.M., Navarro V.M., Fernández-Fernández R., Nogueiras R., Tovar S., Roa J., Vazquez M.J., Vigo E., Casanueva F.F., Aguilar E. (2005). Changes in hypothalamic KiSS-1 system and restoration of pubertal activation of the reproductive axis by kisspeptin in undernutrition. Endocrinology.

[50] Saben J.L., Asquar Z., Rhee J.S., Drury A., Scheaffer S., Moley H. (2016). Excess maternal fructose consumption increases fetal loss and impairs endometrial decidualization in mice. Endocrinology.

[51] Tobiansky D.J., Kachkovski G.V., Enos R.T., Schmidt K.L., Murphy E.A., Soma K.K. (2020). Sucrose consumption alters steroid and dopaminę signalling in the female rat brain. J. Endocrinol..

[52] Niswender G.D., Juengel J.L., Silva P.J., Rollyson M.K., McIntush E.W. (2000). Mechanisms controlling the function and life span of the corpus luteum. Physiol. Rev..

[53] Sagae S.C., Menezes E.F., Bonfleur M.L., Vanzela E.C., Zacharias P., Lubaczeuski C., Franci C.R., Sanvitto G.L. (2012). Early onset of obesity induces reproductive deficits in female rats. Physiol. Behav..

[54] Bowen-Shauver J.M., Gibori G. (2004). The Corpus Luteum of Pregnancy.

[55] Lund S.A., Van Kirk E.A., Murdoch W.J. (1999). Mitogenic and antioxidant mechanisms of estradiol action in preovulatory ovine follicles: Relevance to luteal function. Biol. Reprod..

[56] Günzel-Apel A.U.C., Wolf K., Einspanier A., Oei C., Piechotta M. (2012). Serum progesterone in pregnant bitches supplemented with progestin because of expected or suspected luteal insufficiency. Reprod. Domest. Anim..

[57] Lu J., Wang Z., Cao J., Chen Y., Dong Y. (2018). A novel and compact review on the role of oxidative stress in female reproduction. Reprod. Biol. Endocrinol..

[58] Patterson R.E., Sears D.D. (2017). Metabolic effects of intermittent fasting. Annu. Rev. Nutr..

[59] Sun J., Shen X., Liu H., Peng J., Kuang H. (2021). Caloric restriction in female reproduction: Is it beneficial or detrimental?. Reprod. Biol. Endocrinol..

[60] Chalvon-Demersay T., Blachier F., Tome D., Blais A. (2017). Animal models for the study of the relationships between diet and obesity: A focus on dietary protein and estrogen deficiency. Front. Nutr..

[61] Even P.C., Virtue S., Morton N.M., Fromentin G., Semple R.K. (2017). Editorial: Are rodent models fit for investigation of human obesity and related diseases?. Front. Nutr..

