# Supplementary material for: The Effect of Alternating High-Sucrose and Sucrose Free-Diets, and Intermittent One-Day Fasting on the Estrous Cycle and Sex Hormones in Female Rats
Source: Nutrients. 2022 Oct 17;14(20):4350. doi: 10.3390/nu14204350 (PMC9611605; doi:10.3390/nu14204350)
Supplement: Supplementary file 1 [file nutrients-14-04350-s001.zip › nutrients-1915142-supplementary.pdf]

Supplementary Materials:

Table S1. Component composition of diets.

Component	Basic feed (BF)	Modified feed 1 (MF1)	Modified feed 2 (MF2)
Wheat (g/100 g)	36.4	6.01	6.01
Corn grain (g/100 g)	20.0	10.0	10.0
Wheat bran (g/100 g)	20.0	20.0	20.0
Dry whey (g/100 g)	3.0	3.0	3.0
Fodder salt[1] (g/100 g)	0.3	0.3	0.3
Soya-bean grain (g/100 g)	17.0	17.0	17.0
Fodder chalk[2] (g/100 g)	1.5	1.5	1.5
Phosphate 2-CA[3] (g/100 g)	0.8	0.8	0.8
Premix LRM[4] (g/100 g)	1.0	1.0	1.0
Wheat flour (g/100 g)	0.0	32.4	24.4
Sucrose (g/100 g)	0.0	8.0	16.0

[1]-Mainly NaCl; [2]- Mainly $CaCO_3$; [3]- $CaHPO_4$; [4]- Vitamin-mineral composition used in animals feeds content per kg: IU: A 1500000, vit. D3, 100000; mg: vit. E 8000; vit. K 300, vit. B1 1200, vit. B2 1200, vit. B6 1000, vit. B12 8, Se 100, Fe 16000, Mn 4500, Zn 6000, Cu 1300, I 100, Co 200.

Table S2. Chemical composition of diets.

Component	Basic feed (BF)	Modified feed 1 (MF1)	Modified feed 2 (MF2)
Total protein (%)	17.9 ±0.09	18.4 ±0.12	16.9 ±0.10
% of diet energy	21.0	21.4	19.1
Crude fat (%)	3.2 ±0.11	3.5 ±0.15	3.6 ±0.09
% of diet energy	8.58	9.28	9.05
Carbohydrates (%)			
total	63.1 ±0.55	63.9 ±0.19	65.8 ±0.48
fiber	6.0 ±0.15	4.4 ±0.21	4.4 ±0.13
digested	57.1 ±0.50	59.5 ±0.61	61.5 ±0.44
% of diet energy	70.4	69.3	71.8
Dry matter (%)	90.8 ±0.20	92.4 ±0.16	92.9 ±0.11
Total ash (%)	6.5 ±0.12	6.6 ±0.09	6.6 ±0.18
Metabolizable energy			
(kcal/100 g)	341	352	354
(kJ/100 g)	1425	1471	1484

Table S3. The course of the estrous cycle in females from the control group (CG).

Animal \ Day	1	2	3	4	5	6	7	8	9	10	11	12	13	14	15	16	17	18	19	20	21	Estrus days
1 CG	D	D	E	E	D	D	E	E	D	D	P	E	D	D	D	E	E	D	D	P	E	8
2 CG	D	D	P	E	E	D	D	D	E	E	D	D	D	E	E	D	D	P	E	E	D	8
3 CG	M	D	D	P	E	E	D	D	D	E	E	D	D	P	E	E	E	D	D	P	E	8
4 CG	D	D	D	E	M	D	D	D	E	E	D	D	D	E	D	D	P	E	D	D	D	5
5 CG	D	D	P	E	D	D	D	E	D	D	E	E	D	D	D	E	E	D	D	D	E	6
6 CG	M	D	D	E	E	D	D	D	E	E	D	D	P	E	E	D	D	D	E	E	M	8
7 CG	D	D	E	E	D	D	D	E	E	D	D	P	E	E	D	D	D	E	E	M	D	8
8 CG	M	D	D	E	E	E	D	D	P	E	E	M	D	D	E	E	E	D	D	E	E	9
9 CG	M	D	D	E	E	D	D	D	E	E	D	D	E	E	M	D	D	E	E	E	D	9
10 CG	M	D	D	E	E	D	D	D	E	E	D	D	E	E	D	D	P	E	E	D	D	8

CG – Control group; D – Diestrus; P – Proestrus; E – Estrus; M – Metestrus

Table S4. The course of the estrous cycle in females from the sugar balanced group (SBG).

Animal \ Day	1	2	3	4	5	6	7	8	9	10	11	12	13	14	15	16	17	18	19	20	21	Estrus days
1 SBG	M	D	E	E	E	D	D	D	E	E	D	D	D	E	E	M	D	D	D	P	E	8
2 SBG	D	D	P	E	E	D	D	D	D	P	E	D	D	D	E	E	D	D	D	P	E	6
3 SBG	M	D	E	E	D	D	D	E	M	D	D	D	E	E	D	D	D	E	E	D	D	7
4 SBG	D	D	E	E	E	D	D	E	E	D	D	D	P	E	D	D	D	E	E	M	D	8
5 SBG	M	D	D	D	E	E	D	D	D	E	D	D	D	P	E	M	D	D	D	E	E	6
6 SBG	M	D	P	E	D	E	D	D	E	D	E	M	D	D	P	E	D	D	E	D	P	6
7 SBG	M	D	P	E	D	D	E	D	D	P	E	D	D	E	D	D	P	E	D	D	E	6
8 SBG	D	D	E	M	D	D	E	D	D	D	E	D	D	P	E	E	D	D	D	E	M	6
9 SBG	M	D	E	M	D	D	E	D	D	E	M	D	D	E	M	D	D	E	D	D	E	6
10 SBG	D	D	D	E	D	E	D	D	E	E	D	D	E	D	D	P	E	M	D	D	E	7

SBG – Sugar balanced group; D – Diestrus; P – Proestrus; E – Estrus; M – Metestrus

Table S5. The course of the estrous cycle in females from the high sugar group (HSG).

Day / Animal	1	2	3	4	5	6	7	8	9	10	11	12	13	14	15	16	17	18	19	20	21	Estrus days
1 HSG	D	D	E	D	D	D	P	E	D	D	E	M	D	D	D	D	E	D	D	D	E	5
2 HSG	D	D	E	M	P	E	D	D	E	M	D	D	E	M	P	D	D	E	M	E	E	7
3 HSG	M	D	E	M	D	D	D	E	D	D	D	P	E	D	D	D	E	M	D	D	P	4
4 HSG	E	E	E	D	D	P	E	D	E	M	D	D	P	M	D	D	D	E	D	D	D	6
5 HSG	M	E	E	M	D	D	P	E	M	D	D	D	E	E	D	D	D	P	M	D	D	5
6 HSG	M	D	D	E	E	D	D	D	E	E	D	D	D	P	E	D	D	D	E	E	M	7
7 HSG	D	E	P	D	D	P	E	D	D	D	M	D	D	D	P	E	M	D	D	D	E	4
8 HSG	M	E	P	E	E	D	E	D	P	E	D	D	M	D	D	E	M	M	D	D	P	6
9 HSG	D	E	E	E	D	D	D	D	P	M	E	D	D	P	P	M	D	D	D	E	E	6
10 HSG	E	D	E	D	D	D	P	E	M	D	D	D	E	M	D	D	D	P	E	E	E	7

HSG – High sugar group; D – Diestrus; P – Proestrus; E – Estrus; M – Metestrus

Table S6. The course of the estrous cycle in females from the alternately fed group (AFG).

Day / Animal	1	2	3	4	5	6	7	8	9	10	11	12	13	14	15	16	17	18	19	20	21	Estrus days
1 AFG	D	D	E	E	D	D	D	P	E	D	D	E	M	D	D	D	E	D	D	P	E	6
2 AFG	D	D	D	P	E	D	D	D	D	M	D	D	D	E	E	D	D	P	E	E	M	5
3 AFG	M	D	D	E	D	E	D	D	P	E	M	D	D	E	E	D	D	D	E	M	D	6
4 AFG	D	D	D	E	E	D	D	P	E	D	D	D	E	E	D	D	D	E	M	D	D	6
5 AFG	D	E	M	D	D	E	D	D	E	D	D	D	E	M	D	D	P	E	D	D	E	6
6 AFG	D	D	D	P	E	D	D	D	E	M	D	D	D	D	E	D	D	P	E	M	D	4
7 AFG	M	D	E	D	D	P	E	D	D	D	E	M	D	D	D	E	E	D	D	D	E	6
8 AFG	M	D	E	D	E	D	D	P	E	D	D	E	M	D	D	P	E	D	D	D	E	6
9 AFG	E	D	D	E	E	E	D	D	P	M	D	D	D	E	D	D	D	P	E	D	D	6
10 AFG	P	E	M	E	D	D	D	P	M	D	D	D	E	E	D	D	D	P	E	M	D	5

AFG – Alternately fed group; D – Diestrus; P – Proestrus; E – Estrus; M – Metestrus

Table S7. The course of the estrous cycle in females from the periodically starved group (PSG).

Day / Animal	1	2	3	4	5	6	7	8	9	10	11	12	13	14	15	16	17	18	19	20	21	Estrus days
1 PSG	D	D	D	E	E	D	D	P	E	M	D	D	D	E	D	D	D	E	M	D	D	5
2 PSG	D	P	E	D	D	D	P	E	M	D	D	D	E	D	D	D	M	D	D	D	E	4
3 PSG	M	P	D	D	P	E	D	D	E	M	D	D	D	E	E	M	D	D	M	D	P	4
4 PSG	D	D	D	E	E	D	D	D	M	D	D	D	E	D	D	D	P	E	M	D	D	4
5 PSG	M	D	D	D	P	D	D	P	E	D	D	D	E	M	D	D	D	E	D	D	P	3
6 PSG	D	M	D	D	D	D	E	D	D	D	P	E	M	D	D	E	D	D	D	P	E	4
7 PSG	M	D	D	D	P	E	D	D	D	E	M	D	D	P	M	D	D	E	E	D	D	4
8 PSG	D	E	D	P	E	D	D	E	D	D	P	E	D	D	D	E	M	D	D	E	M	6
9 PSG	D	P	E	E	D	D	D	P	M	D	E	E	M	P	E	D	D	D	D	E	M	6
10 PSG	M	E	E	D	P	D	D	D	E	M	D	P	M	D	D	D	E	D	D	D	P	4

PSG – Periodically starved group; D – Diestrus; P – Proestrus; E – Estrus; M – Metestrus

Table S8. P-values for the statistics of hormone concentration determinations.

Hormone	P-value
**LH**	CG *vs* HSG 0.001; CG *vs* AFG 0.002; CG *vs* PSG 0.0009<br>SBG *vs* HSG 0.001; SBG *vs* AFG 0.001; SBG *vs* PSG 0.0007<br>HSG *vs* PSG 0.036<br>AFG *vs* PSG 0.034
**FSH**	CG *vs* PSG 0.029<br>SBG *vs* PSG 0.030<br>HSG *vs* PSG 0.029<br>AFG *vs* PSG 0.029
**E2**	CG *vs* SBG 0.0001; CG *vs* HSG 0.0002; CG *vs* AFG 0.0002; CG *vs* PSG 0.0001<br>SBG *vs* PSG 0.049<br>HSG *vs* PSG 0.048<br>AFG *vs* PSG 0.048
**Pg**	CG *vs* SBG 0.048; CG *vs* HSG 0.001; CG *vs* AFG 0.002; CG *vs* PSG 0.001<br>SBG *vs* HSG 0.002; SBG *vs* AFG 0.003; SBG *vs* PSG 0.001<br>HSG *vs* PSG 0.049<br>AFG *vs* PSG 0.049
**T**	CG *vs* AFG 0.049<br>SBG *vs* AFG 0.047<br>HSG *vs* AFG 0.048<br>AFG *vs* PSG 0.047
**SHGB**	CG *vs* HSG 0.043; CG *vs* AFG 0.045<br>SBG *vs* HSG 0.039; SBG *vs* AFG 0.041<br>HSG *vs* PSG 0.048<br>AFG *vs* PSG 0.049

CG – Control group; SBG – Sugar balanced group; HSG – High sugar group; AFG – Alternately fed group; PSG – Periodically starved group. LH - luteinizing hormone; FSH - follicle stimulating hormone; E2 – estradiol; Pg – progesterone; T – testosterone; SGBD - sex hormone-binding globulin.
